# Supplementary material for: Hyperbolic polaritonic crystals with configurable low-symmetry Bloch modes
Source: Nat Commun. 2023 Jul 1;14:3894. doi: 10.1038/s41467-023-39543-w (PMC10314936; doi:10.1038/s41467-023-39543-w)
Supplement: Supplementary file 1 — Supplementary Information [file 41467_2023_39543_MOESM1_ESM.pdf]

Supplementary Information for

Hyperbolic polaritonic crystals with configurable low-symmetry Bloch modes

Jiangtao Lv,[1,2†] Yingjie Wu,[3†*] Jingying Liu,[4,5†] Youning Gong,[6] Guangyuan Si,[7] Guangwei Hu,[8] Qing Zhang,[9] Yupeng Zhang,[6] Jian-Xin Tang,[4,10] Michael S. Fuhrer,[11,12] Hongsheng Chen,[3] Stefan A. Maier,[11,12,13] Cheng-Wei Qiu,[14*] Qingdong Ou[4,5,11*]

[1]College of Information Science and Engineering, Northeastern University, Shenyang 110004, China.

[2]School of Control Engineering, Hebei Key Laboratory of Micro-Nano Precision Optical Sensing and Measurement Technology, Northeastern University at Qinhuangdao, Qinhuangdao 066004, China.

[3]ZJU-Hangzhou Global Scientific and Technological Innovation Center, Zhejiang University, Hangzhou 311215, China.

[4]Macao Institute of Materials Science and Engineering (MIMSE), Faculty of Innovation Engineering, Macau University of Science and Technology, Taipa, Macao 999078, China.

[5]Department of Materials Science and Engineering, Monash University, Clayton, Victoria 3800, Australia.

[6]State Key Laboratory of Radio Frequency Heterogeneous Integration, College of Electronics and Information Engineering, Shenzhen University, Shenzhen 518060, China.

[7]Melbourne Centre for Nanofabrication, Victorian Node of the Australian National Fabrication Facility, Clayton 3168, VIC, Australia.

[8]School of Electrical and Electronic Engineering, Nanyang Technological University, Singapore 639798, Singapore.

[9]School of Physics, University of Electronic Science and Technology of China, Chengdu 611731, China.

[10]Jiangsu Key Laboratory for Carbon-Based Functional Materials & Devices, Institute of Functional Nano & Soft Materials (FUNSOM), Soochow University, Jiangsu 215123, China.

[11]ARC Centre of Excellence in Future Low-Energy Electronics Technologies, Monash University, Clayton, Victoria 3800, Australia.

[12]School of Physics and Astronomy, Monash University, Clayton, Victoria 3800, Australia.

[13]Department of Physics, Imperial College London, London SW7 2AZ, UK.

[14]Department of Electrical and Computer Engineering, National University of Singapore, Singapore 117583, Singapore.

[†]These authors contributed equally to this work.

[*]Correspondence to: yingjie.wu@zju.edu.cn (Y.W.); chengwei.qiu@nus.edu.sg (C.-W.Q.); qdou@must.edu.mo (Q.O.)

Supplementary Note 1. Scaling law of PoCs.

The scaling law in Fig. 1 was obtained by combining numerical simulation and fitting. Using the one for hyperbolic PoCs as an example, we first conducted numerical simulations to obtain the absorption spectra of PoCs with varied periodicies at certain directions. Resonance frequencies ($\omega_j^r, j = x, y$) were then extracted and compared with that of the square-type PoC with $P_x = P_y = P_0$ (ω_0^r) to obtained frequency shifts $\Delta\omega_j^r = \omega_j^r - \omega_0^r$. Here we just focused on the strongest polariton resonant absorption peak instead of the periodicity-independent phonon absorption peak, as shown in Supplementary Figure 1a.

We then fitted the numerical data based on the Rayleigh-Wood anomaly. At this condition,

$$\mathbf{k}_p = \mathbf{k}_\parallel + m\mathbf{G}_x + n\mathbf{G}_y \tag{1}$$

where $\mathbf{k_p} = \sqrt{\mathbf{k}_x^2 + \mathbf{k}_y^2}$ and $\mathbf{k}_\parallel$ represent the in-plane polariton wavevector and in-plane incident wavevector; $\mathbf{G_x} = \frac{2\pi}{P_x}\hat{x}$ and $\mathbf{G_y} = \frac{2\pi}{P_y}\hat{y}$ are reciprocal lattice vectors for the periods P_x and P_y; m and n are diffraction orders. For normal incidence, $\mathbf{k}_\parallel = 0$, and the resonance frequency is solved to be $\omega_x^r = \frac{c}{\sqrt{\varepsilon_x^{eff}}}\frac{m}{P_x}$ at the x direction and $\omega_y^r = \frac{c}{\sqrt{\varepsilon_y^{eff}}}\frac{n}{P_y}$ at the y direction. Here we calculate through the dispersion relation of surface polaritons $\mathbf{k_p} = \frac{2\pi\omega}{c}\sqrt{\varepsilon_{eff}}$ where ε_{eff} is the effective permittivity. For volume-confined polaritons, they can also be treated as two-dimensional surfaces with effective conductivities. Finally, we can reach the equation for the frequency shift:

$$\Delta\omega_x^r = \frac{mc}{\sqrt{\varepsilon_x^{eff}}}\left(\frac{1}{P_x} - \frac{1}{P_0}\right) = \frac{mc}{\sqrt{\varepsilon_x^{eff}}}\frac{P_0 - P_x}{P_x P_0} \tag{2}$$

for the x-packed arrays, and

$$\Delta\omega_y^r = \frac{nc}{\sqrt{\varepsilon_y^{eff}}}\left(\frac{1}{P_y} - \frac{1}{P_0}\right) = \frac{nc}{\sqrt{\varepsilon_y^{eff}}}\frac{P_0 - P_y}{P_y P_0} \tag{3}$$

for the y-packed arrays.

As $\frac{mc}{P_0\sqrt{\varepsilon_x^{eff}}}$ and $\frac{nc}{P_0\sqrt{\varepsilon_y^{eff}}}$ are independent on P_x and P_y, we used a simple function $y = A(1 - x)/x$ to fit the numerical results and found a good agreement in Supplementary Figure 1b.

We used this method to qualitatively describe the scaling laws of polaritonic crystals made from polaritonic materials with different permittivities and in-plane anisotropy. The normalized fitting curves were illustrated in Fig. 1.

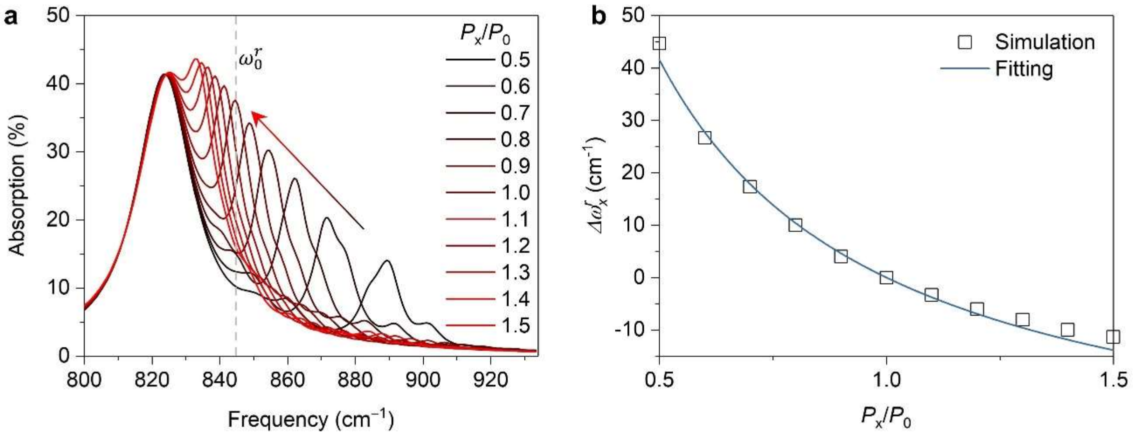

Supplementary Figure 1. **a**, Calculated absorption spectra of hyperbolic PoCs with a fixed P_y but varied P_x. **b**, Extracted frequency shifts and fitting results.

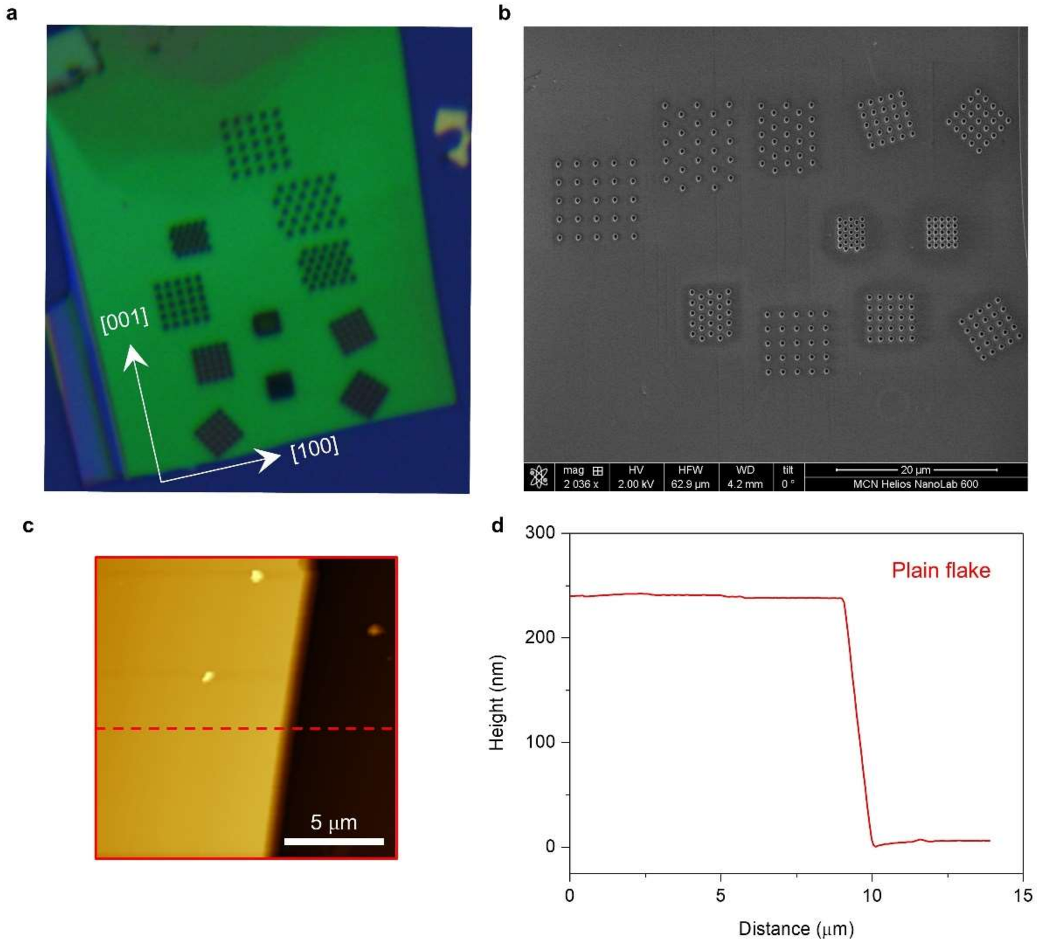

Supplementary Figure 2. **a**, Optical image of prepared α-MoO_3 slab and hyperbolic PoCs. **b**, SEM image. **c**, AFM image around the edge of the flake. **d**, Height profile extracted along the red dashed line in **c**.

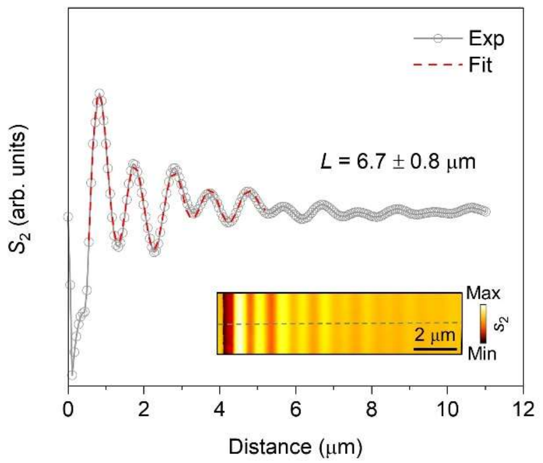

Supplementary Figure 3. Extraction of propagation length (L). We conducted near-field measurement at 892 cm^{-1} on the same flake but near the edge where hole arrays are absent. The extracted line trace along the gray dashed line in the near-field amplitude image (inset) was fitted using the equation $s(x) = A\frac{e^{\frac{-2x}{L}}}{\sqrt{x}}\sin\frac{4\pi(x-x_c)}{\lambda_p} + B\frac{e^{\frac{-x}{L}}}{x}\sin\frac{2\pi(x-x_c')}{\lambda_p}$, where A and B are the parameters for tip- and edge-launched PhPs, x is the distance from edge, x_c and x_c' are phase shifts, λ_p is polariton wavelength. A propagation length of 6.7 ± 0.8 μm was obtained.

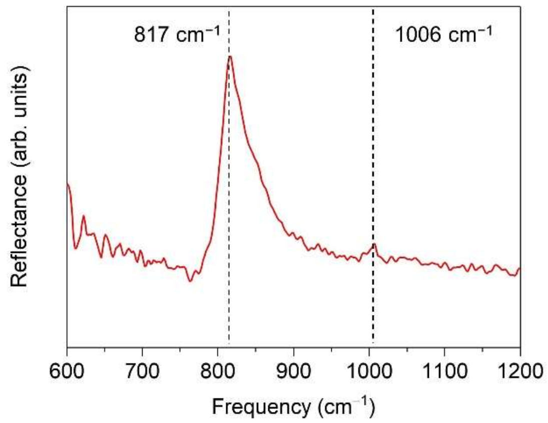

Supplementary Figure 4. Reflectance spectra of the PoCs composed of 5×5 hole arrays with P = 2.3 μm.

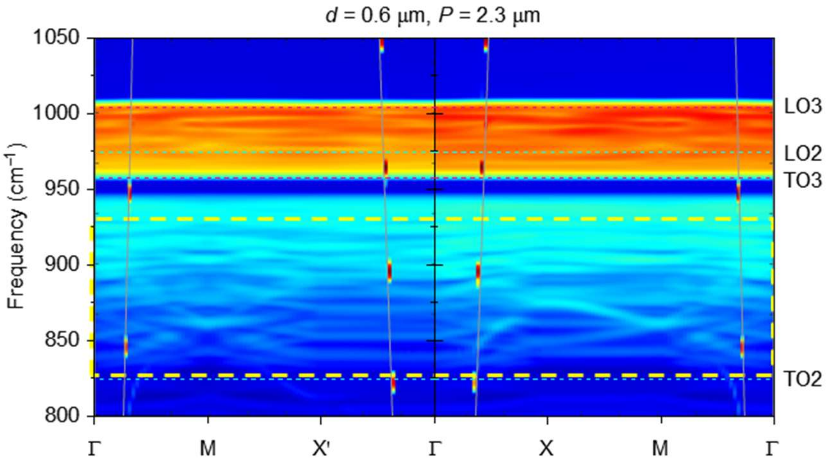

Supplementary Figure 5. Band structure of the PoC with $P = 2.3$ μm. The gray curves represent light lines. Yellow dashed rectangle sorrounds the frequency domain considered in the main text.

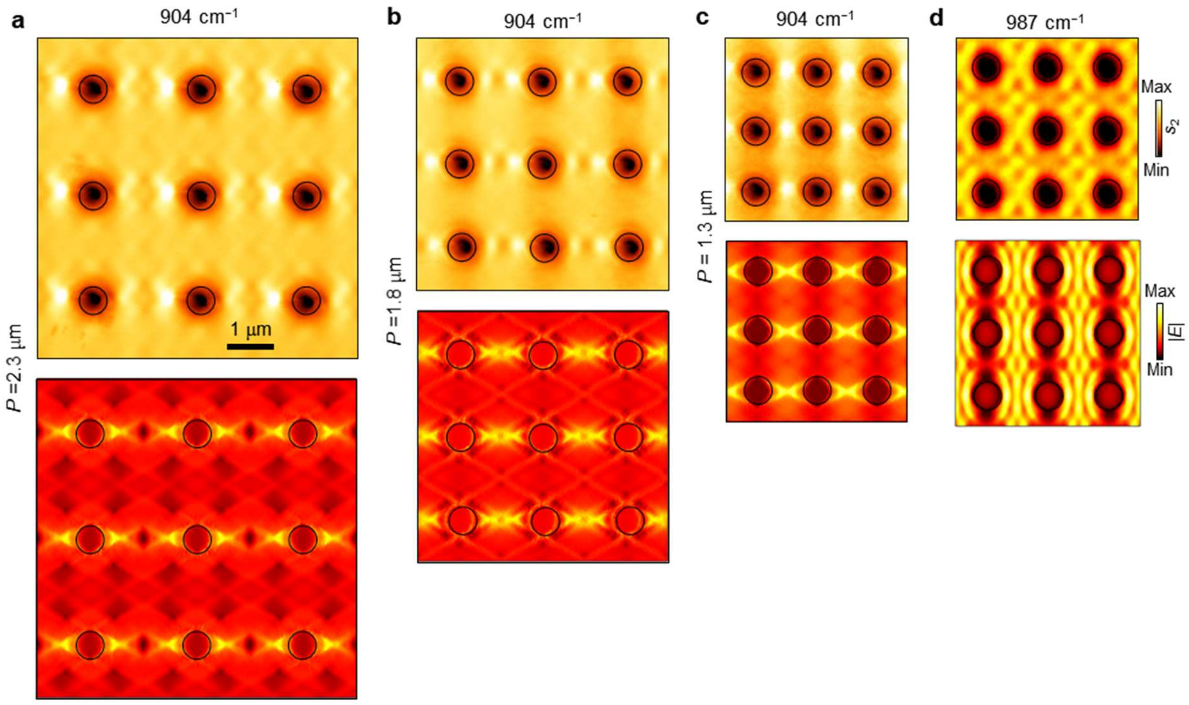

Supplementary Figure 6. Near-field interference patterns and corresponding field distribution images of the PoC with $P = 2.3$ μm at 904 cm^{-1} (**a**), $P = 1.8$ μm at 904 cm^{-1} (**b**), the PoC with $P = 1.3$ μm at 904 cm^{-1} (**c**) and 987 cm^{-1} (**d**).

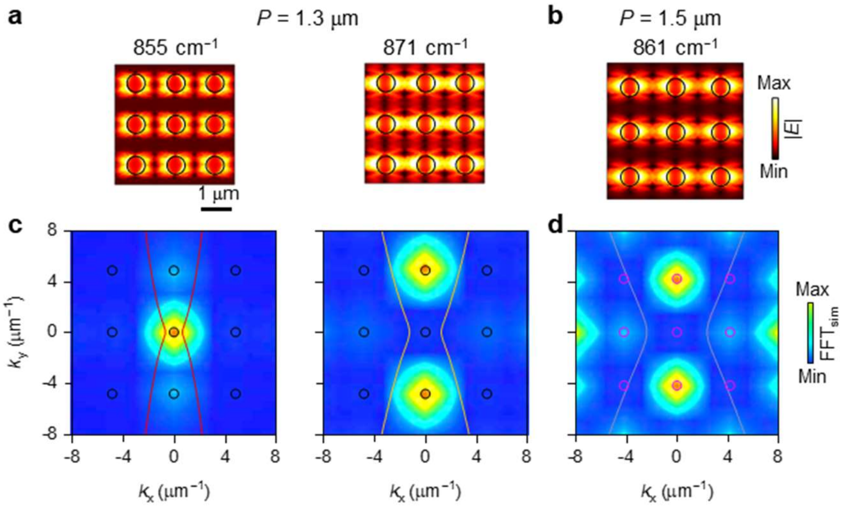

Supplementary Figure 7. Simulated electric field distribution images (top) and corresponding FFT maps (bottom) of PoCs with P = 1.3 (**a**) and 1.5 μm (**b**) at resonance frequencies. Coloured curves in (**c**) and (**d**) represent IFC contours, respectively. Black and pink circles indicate reciprocal space points.

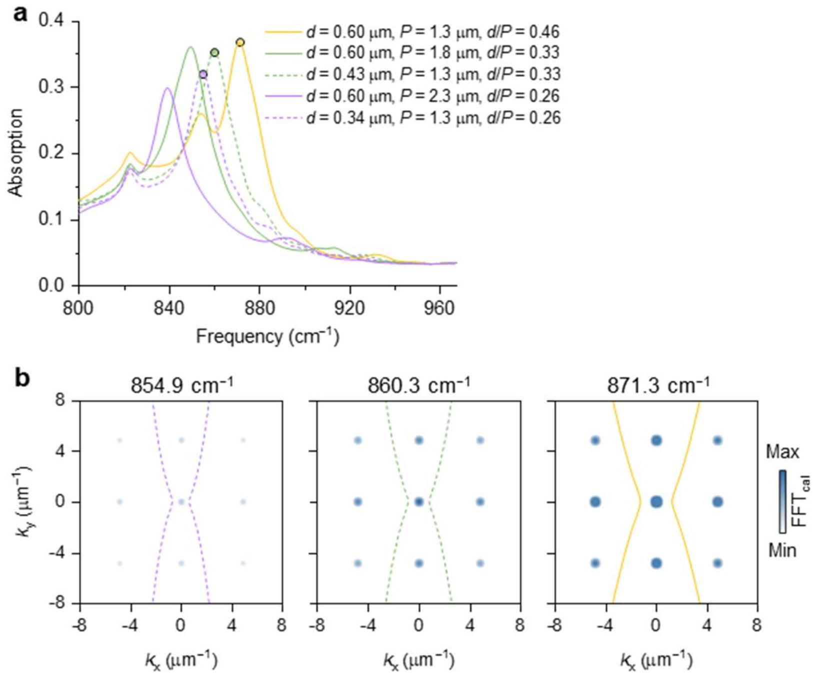

Supplementary Figure 8. **a**, Absorption spectra of PoCs with varied d/P ratios. **b**, IFCs and normalized FFT amplitude maps at the conditions marked by coloured dots in **a**.

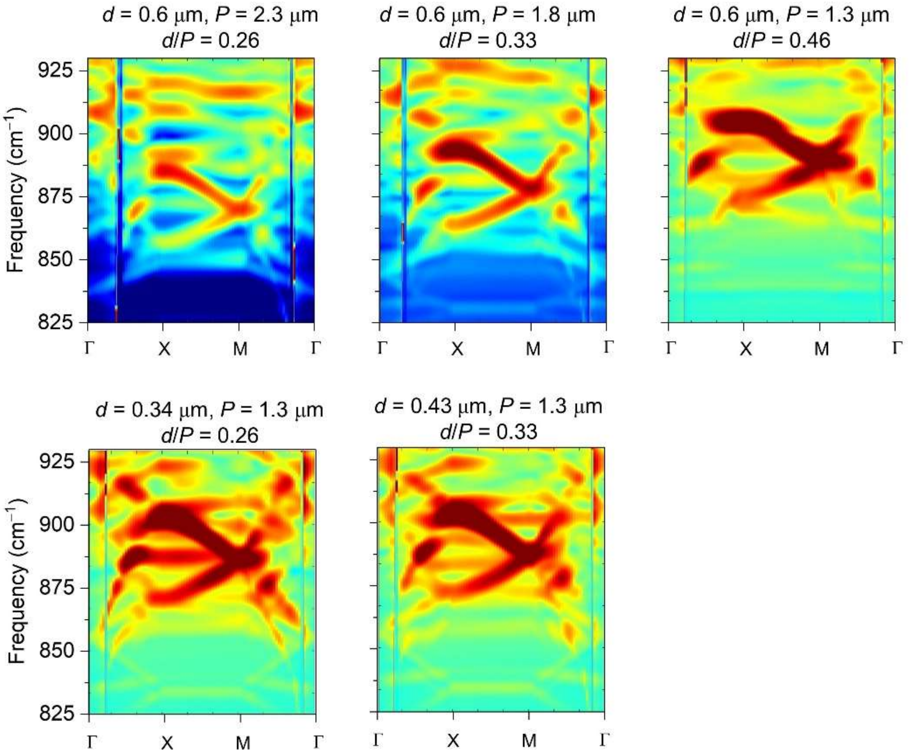

Supplementary Figure 9. Band structures of PoCs with varied diameters and periodicities. The map intensity was normalized. The gray curces represent light lines at certain periodicities.

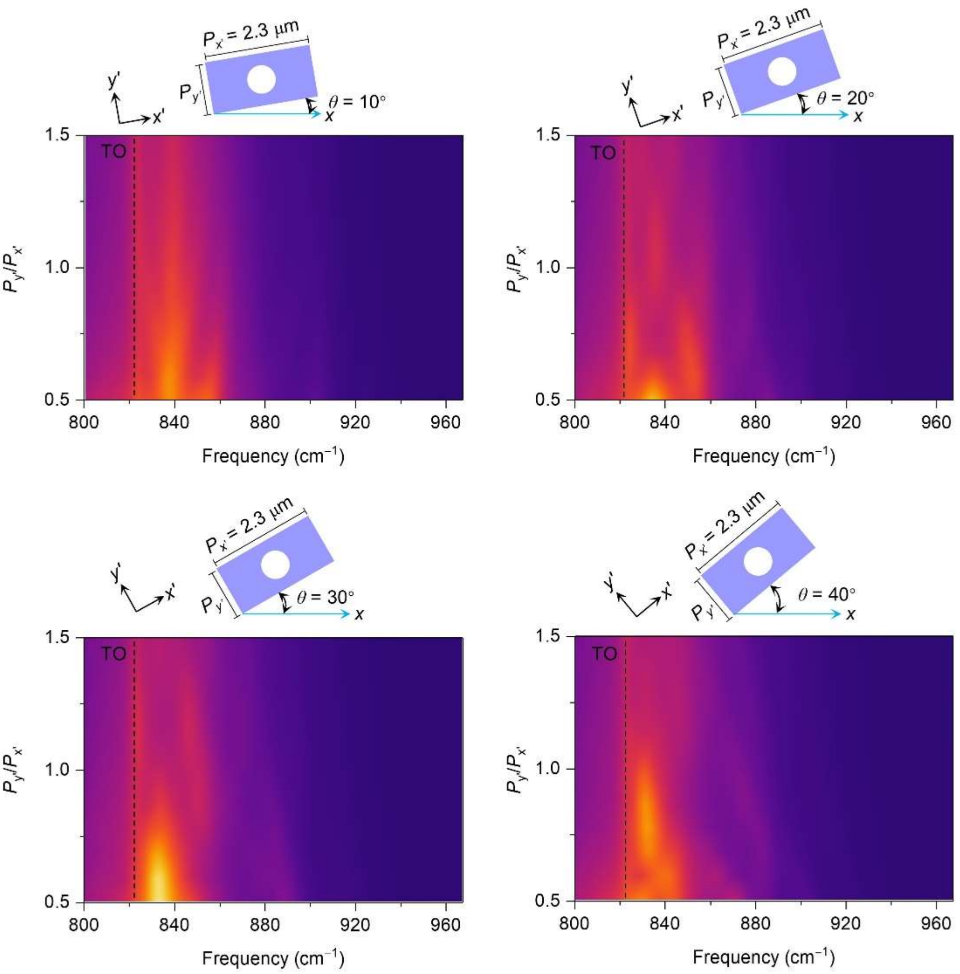

Supplementary Figure 10. Calculated absorption coefficients of the PoCs tilted by different angles as a function of frequency and length-width ratio.
